# Supplementary material for: Coordinated hedgehog signaling induces new hair follicles in adult skin
Source: eLife. 2020 Mar 17;9:e46756. doi: 10.7554/eLife.46756 (PMC7077985; doi:10.7554/eLife.46756)
Supplement: Transparent reporting form [file elife-46756-transrepform.docx]

***eLife’s* transparent reporting form**

We encourage authors to provide detailed information *within their submission* to facilitate the interpretation and replication of experiments. Authors can upload supporting documentation to indicate the use of appropriate reporting guidelines for health-related research (see [EQUATOR Network](http://www.equator-network.org/%20)), life science research (see the [BioSharing Information Resource](https://biosharing.org/)), or the [ARRIVE guidelines](http://www.plosbiology.org/article/info:doi/10.1371/journal.pbio.1000412) for reporting work involving animal research. Where applicable, authors should refer to any relevant reporting standards documents in this form.

If you have any questions, please consult our Journal Policies and/or contact us: [editorial@elifesciences.org](mailto:editorial@elifesciences.org).

**Sample-size estimation**

- You should state whether an appropriate sample size was computed when the study was being designed
- You should state the statistical method of sample size computation and any required assumptions
- If no explicit power analysis was used, you should describe how you decided what sample (replicate) size (number) to use

Please outline where this information can be found within the submission (e.g., sections or figure legends), or explain why this information doesn’t apply to your submission:

We use animal models leading to a clear phenotype dependent on the genotype. We used a minimum of three animals per genotype and time point, and the details are provided in the “List of mouse experiments” (Table S1).

**Replicates**

- You should report how often each experiment was performed
- You should include a definition of biological versus technical replication
- The data obtained should be provided and sufficient information should be provided to indicate the number of independent biological and/or technical replicates
- If you encountered any outliers, you should describe how these were handled
- Criteria for exclusion/inclusion of data should be clearly stated
- High-throughput sequence data should be uploaded before submission, with a private link for reviewers provided (these are available from both GEO and ArrayExpress)

Please outline where this information can be found within the submission (e.g., sections or figure legends), or explain why this information doesn’t apply to your submission:

We used at least three biological replicates (i.e. independent mice) per genotype and main time points of our study (10 days, 5 weeks and 9 weeks post tamoxifen treatment). For every animal, numerous TDs were analyzed, depending on size of tissue sample (from 3-5 TDs for biopsies to 10-15 TDs for final material). Additionally, to cover development stages of de novo HF of Gli1^creERT2^;R26^Tom^;Ptch1^fl/fl^ we analyzed 5 mice between T27d and T36d both in dorsal skin and in paws. Most of the experiments (including technical replicates of antibody stainings, animal treatments and resulting phenotype assessment, and data-point counting and analyses) were performed independently by multiple researchers within the lab.

The number of mice is given in the figure legends. When giving all details in the main text or figure legend would be too exhaustive, the details are given in the methods (see “Measurements and quantitative image analysis of de novo hair follicles”; for Figure 2C). All details are provided in the “List of mouse experiments” (Table S1).

We did not encounter or remove outliers. Importantly, we even present very rare observations (2 hairs observed in Figure 1I, Lgr6 model) and describe and discuss this observation in the text as we think such information is important in general and to this study in particular.

The study does not contain sequencing data.

**Statistical reporting**

- Statistical analysis methods should be described and justified
- Raw data should be presented in figures whenever informative to do so (typically when N per group is less than 10)
- For each experiment, you should identify the statistical tests used, exact values of N, definitions of center, methods of multiple test correction, and dispersion and precision measures (e.g., mean, median, SD, SEM, confidence intervals; and, for the major substantive results, a measure of effect size (e.g., Pearson's r, Cohen's d)
- Report exact p-values wherever possible alongside the summary statistics and 95% confidence intervals. These should be reported for all key questions and not only when the p-value is less than 0.05.

Please outline where this information can be found within the submission (e.g., sections or figure legends), or explain why this information doesn’t apply to your submission:

Quantification approach and statistical analysis methods are described in ‘Materials and Methods’ as a separate section named ‘*Measurements and quantitative image analysis of de novo hair follicles’.*

For quantification, we also provide the statistical test and p-values in the figure legend (Figure 2C and Figure 8D). Additionally, we provide supplementary source data tables containing the raw data used for quantifications.

(For large datasets, or papers with a very large number of statistical tests, you may upload a single table file with tests, Ns, etc., with reference to sections in the manuscript.)

**Group allocation**

- Indicate how samples were allocated into experimental groups (in the case of clinical studies, please specify allocation to treatment method); if randomization was used, please also state if restricted randomization was applied
- Indicate if masking was used during group allocation, data collection and/or data analysis

Please outline where this information can be found within the submission (e.g., sections or figure legends), or explain why this information doesn’t apply to your submission:

We used litter mates where available for all experimental groups, including males and females. No further randomization was performed. Masking (or blinding) was not applicable in our study due to the obvious phenotypes of the mice with different genotypes. Nevertheless, as a standard in my research group, we ask researchers not directly involved in experiments to also look at the images or in the microscope and describe what they see (e.g. no/slight/severe changes in cell type and skin compartment), which we also did for this study.

**Additional data files (“source data”)**

- We encourage you to upload relevant additional data files, such as numerical data that are represented as a graph in a figure, or as a summary table
- Where provided, these should be in the most useful format, and they can be uploaded as “Source data” files linked to a main figure or table
- Include model definition files including the full list of parameters used
- Include code used for data analysis (e.g., R, MatLab)
- Avoid stating that data files are “available upon request”

Please indicate the figures or tables for which source data files have been provided:

We provide the source data for Figure 2C (Figure 2-source data 1) and for Figure 8D (Figure 8-source data 1). We also provide Table 1, listing all mouse experiments (genotype, treatment, analysis time point, tissue sampling, observations/phenotype, mouse ID).
